# Glass surface as strong base, ‘green’ heterogeneous catalyst and degradation reagent†

**DOI:** 10.1039/d1sc02708e

**Published:** 2021-06-23

**Authors:** Yangjie Li, Kai-Hung Huang, Nicolás M. Morato, R. Graham Cooks

**Affiliations:** Department of Chemistry, Purdue University West Lafayette IN 47907 USA cooks@purdue.edu

## Abstract

Systematic screening of accelerated chemical reactions at solid/solution interfaces has been carried out in high-throughput fashion using desorption electrospray ionization mass spectrometry and it provides evidence that glass surfaces accelerate various base-catalyzed chemical reactions. The reaction types include elimination, solvolysis, condensation and oxidation, whether or not the substrates are pre-charged. In a detailed mechanistic study, we provide evidence using nanoESI showing that glass surfaces can act as strong bases and convert protic solvents into their conjugate bases which then act as bases/nucleophiles when participating in chemical reactions. In aprotic solvents such as acetonitrile, glass surfaces act as ‘green’ heterogeneous catalysts that can be recovered and reused after simple rinsing. Besides their use in organic reaction catalysis, glass surfaces are also found to act as degradation reagents for phospholipids with increasing extents of degradation occurring at low concentrations. This finding suggests that the storage of base/nucleophile-labile compounds or lipids in glass containers should be avoided.

## Abstract 2

Glass surfaces are found to be strong bases, ‘green’ heterogeneous catalysts and degradation reagents: glass microspheres act as strong bases to accelerate multiple base-catalyzed reaction types by a factor of 26–2021.

## Graphical abstract

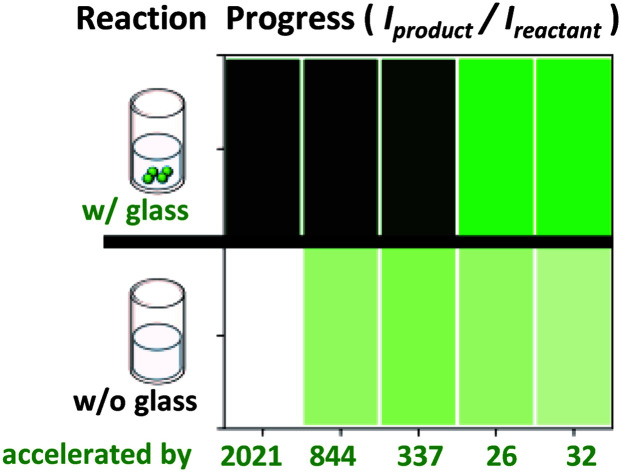

## Introduction

Despite scattered reports on the phenomenon of chemical reactions being affected by glass containers,^1–5^ the process has yet to be fully understood. Systematic study on glass catalyzed chemical reactions is needed to provide experimental evidence to support any proposed mechanism. A recent preliminary communication demonstrated accelerated chemical reactions at glass surfaces in the case of the Katritzky transamination reaction.^6^ This effect was shown in glass containers relative to plastic containers and by elution of glass particles from glass surfaces into the reaction solution. It was validated using glass microspheres of large surface area and the silanolate anions at the glass/solution interface were suggested to act as a base to accelerate the Katritzky reaction by up to two orders of magnitude. The current study aims to investigate the reaction types and substrate scope of this phenomenon so as to gain a deeper understanding of how glass surfaces affect chemical reactions.

In high-throughput experimentation (HTE), large data sets can be generated quickly, facilitating synthetic route screening and pharmaceutical research.^7–12^ Recently, glass beads have been used to deliver nanomoles of solid reagents in high-throughput reaction screening^13^ but, to our best knowledge, there has been no report on studying glass-catalyzed chemical reactions systematically using any high-throughput screening (HTS) system. In this study we use a desorption electrospray ionization mass spectrometry (DESI-MS)^14,15^ based high-throughput system^16^ with hardware and software features that enable reproducible quantitative data to be generated from minimal amounts (50 nL) of sample solution. The integrated automatic platform allows both synthetic reactions^17^ and enzymatic reactions^18^ to be studied at a throughput of 1 s per sample. Therefore, we performed a single-day high-throughput experiment using the HTE DESI-MS system to screen a large set of glass-catalyzed reactions.

HTE DESI-MS screening allows fast comparisons between reactions with and without glass microspheres, but we are aware that the acceleration factor seen in the DESI-MS data will include contributions from droplet acceleration^19,20^ as well as glass catalysis. Therefore, nanoelectrospray ionization mass spectrometry (nESI-MS) was chosen for the subsequent detailed studies considering its superb sensitivity^21–23^ and for the fact that when using a short distance between electrode and MS inlet as well as a small capillary orifice,^24^ nESI-MS can be used as a reliable non-accelerating analytical method.^6,25,26^

Glass is not an inert medium and cells could adhere to freshly formed glass surfaces while bioglass is known to be beneficial to tissue growth in clinical use.^27^ Aside from the use of silica-based materials for containers to store solutions, silica nanoparticles are also an emerging tool in biomedical applications such as drug delivery and imaging.^28,29^ An additional goal of this work was to evaluate the possible role of glass on chemical stability of stored biochemicals, specifically phospholipids. Evaluation of signaling lipids and lipidomics are crucial for understanding biological processes,^30^ and recently a MS-based workflow has been developed to study the impact of storage parameters on lipid stability.^31^ However, there has been a lack of experimental data on how container materials such as glass surfaces might chemically impact the storage of biomolecules in solutions although physical adsorption has been reported.^32^ Such data is indispensable considering current advances in glass manufacturing^33^ which could lead to a much wider use of glass to store drug products such as the COVID-19 vaccines.^34^ Previously, forced degradation of small-molecule pharmaceutics and therapeutic peptides have been studied in detailed in a fast fashion in confined volumes such as levitated droplets.^25,35^ Here, we are especially interested in how glass surfaces could potentially induce chemical degradation of lipids, peptides, and neuromodulators, specifically phospholipids, and glutathione as well as acetylcholine.

Some facts relevant to this study come from the literature of capillary electrophoresis as well as materials science as discussed below. Glass surfaces are covered with dissociable silanol groups and in contact with a solution, the surface will be negatively charged surface and consist of silanolate anions covered by an electric double layer at the solid/solution interface due to the preferential adsorption and attraction of ions onto and close to the surface.^36^ Aprotic solvents such as acetonitrile (ACN) can only accept but not donate protons. These solvents can be protonated by surface silanol groups to form silanolate but not deprotonated; amphiprotic solvents such as methanol (MeOH) and water (H_2_O) yield both protonated and deprotonated solvent molecules by auto-ionization. As a result of the low concentrations of cations in acetonitrile, there is much less shielding of the negative charge at the surface in this solvent.^37^ In addition, the p*K*_a_ value of silanol groups is the largest in acetonitrile, followed by methanol, and the smallest in water where both protons and silanolate anions can be stabilized by solvation. Therefore, the base strength of silanolate groups is highest in acetonitrile and the magnitude of ionic adsorption on the silica surface will vary due to differences in stabilization of ions in different solvents.^38^ It is known in materials science that the structure of soda lime glass can vary from the typical composition shown in the Experimental section. This is described by the so-called modified random network where at the nano-scale the sodium ions are non-uniformly distributed within the glass and so on its surface.^39^ Moreover, mass spectrometric analysis of newly formed surfaces revealed significant amounts of alkali ions compared to the initial glass surface.^40^

## Results and discussion

### HTS of glass-promoted chemical reactions by DESI-MS

**Fig. 1:**
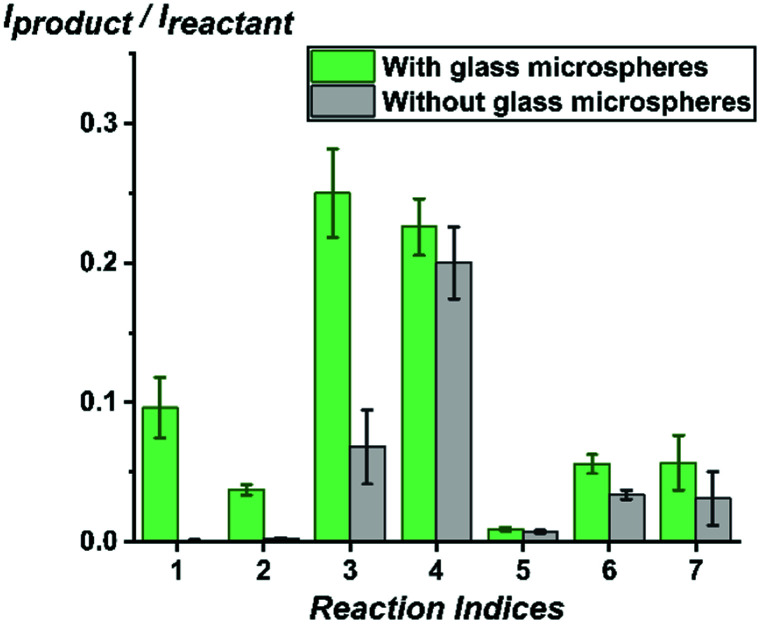
Comparison of reaction progress with glass microspheres (0.02 eq. silanolate groups, estimated) and without glass microspheres analysed by DESI-MS (average of 16 replicate analyses). Significant differences in reaction kinetics were found for the seven chemical reactions studied: (1) elimination of HCl from 3-chloro-2-hydroxypropyl trimethylammonium; (2) solvolysis of acetylcholine to choline; (3) imine formation between Girard's reagent T and 2-pyridinecarbaldehyde; (4) Katritzky reaction between 2,4,6-triphenylpyrylium and *p*-anisidine; (5) Katritzky reaction between 2,4,6-triphenylpyrylium and Girard's reagent T; (6) Knoevenagel condensation between 1,2,3,3-tetramethyl-3*H*-indolium and 2-pyridinecarbaldehyde; (7) Knoevenagel condensation between 1,2,3,3-tetramethyl-3*H*-indolium and 3-hydroxybenzaldehyde.

First, we used the high-throughput system which is capable of screening thousands of reaction conditions per hour^16–18^ to test how various base-catalyzed chemical reactions, including elimination, solvolysis, imine formation, Katritzky reaction and Knoevenagel condensation, are affected by addition of glass microspheres. Methanol was added to containers containing glass microspheres (diameter: 32.5 μm, 0.02 equivalents) and then reactant solutions in methanol were added in stoichiometric amounts to reach a final concentration of 1 mM. Right after mixing at room temperature, aliquots were sampled from the solution without further incubation or disturbance of the glass microspheres at the bottom. Using a Biomek i7 liquid handling robot, 50 nL of solutions from each sample were pinned on a plastic slide glued on a glass backing. A stream of charged DESI droplets releases secondary droplets, so accelerating reactions in the mixture pinned on the surface: DESI-MS is not simply an ambient ionization method^15^ for analysis of reaction mixtures^41–43^ it also accelerates chemical reactions in microdroplets.^19^ As shown in Fig. 1, significant differences in reaction kinetics were found between reactions with glass microspheres (0.02 eq. silanolate groups estimated using the so-called Kiselev–Zhuravlev constant,^44,45^ an experimentally measured concentration of 5 OH groups per nm^2^ glass surface) and without glass microspheres added in the reaction mixture in methanol for the seven chemical reactions. Notably, the presence of glass microspheres turned some reactions such as E_2_ elimination and solvolysis from ‘NO’ to ‘YES’ reactions under the studied conditions.

### Glass-promoted chemical reactions studied by nESI-MS

Encouraged by the success in screening multiple reactions whose kinetics were enhanced by glass microspheres, we moved on to study in more detail the scope of the glass effect. To achieve this, we moved from DESI-MS to nESI-MS as a robust and sensitive analytical method which itself does not accelerate chemical reactions.^46^ We chose all three reaction types represented in the methanol data of Fig. 1 and added another so in all, the types were elimination, solvolysis, condensation (two examples), and oxidation (Table 1). We mixed the reactants at 50 μM, a condition shown to accelerate reactions to a great extent at both the air/solution^26,47,48^ and solid/solution interfaces.^6^

**Table 1:** Progress of five reactions showing much higher rates with glass microspheres than without glass microspheres

Reaction types	Reaction schemes^a^	*I*_P_/*I*_R_ (with glass)^b^	*I*_P_/*I*_R_ (without glass)^b^	Accel. factor^c^
(1) Elimination	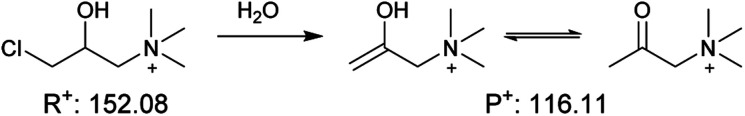	2.9 ± 0.1	0.0014 ± 0.0003	2.0 × 10^3^
(2) Solvolysis	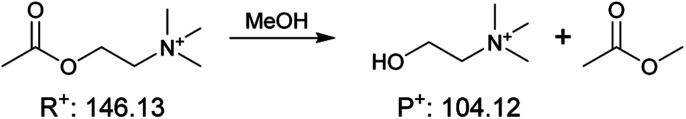	4.0 ± 1.1	0.0047 ± 0.0005	8.4 × 10^2^
(3) Condensation (charged imine)	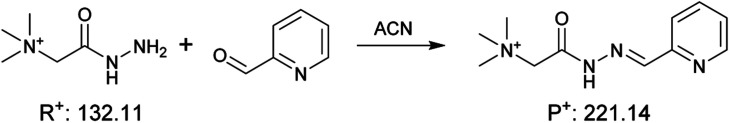	2.0 ± 0.1	0.0058 ± 0.0016	3.4 × 10^2^
(4) Condensation (neutral imine)	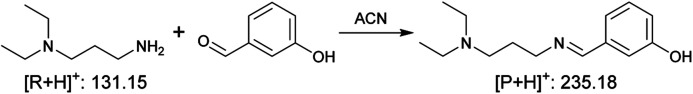	0.11 ± 0.02	0.0042 ± 0.0025	2.6 × 10^1^
(5) Oxidation of thiol to disulfide	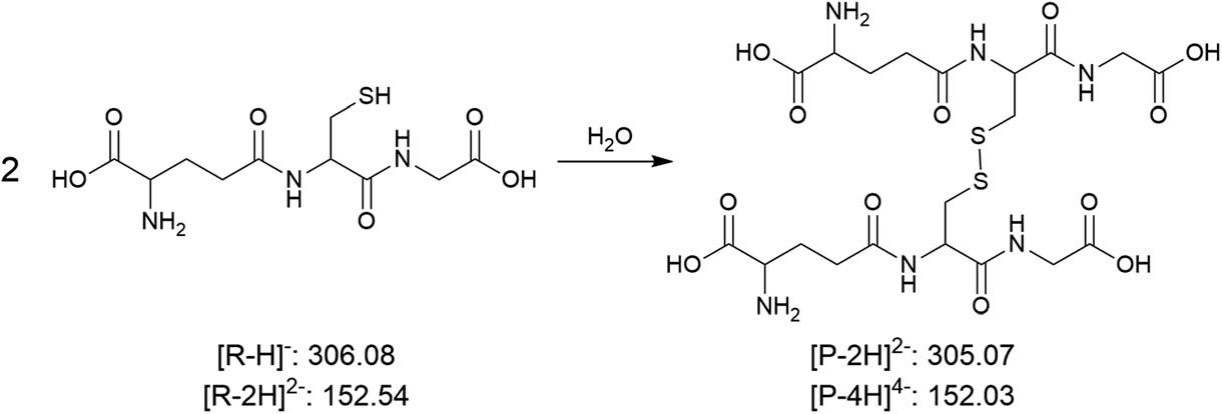	0.11 ± 0.07	0.0033 ± 0.0003	3.2 × 10^1^

a Reactions were performed for 4 h in the specific solvent indicated at 50 μM for each reactant in the scheme with glass microspheres (0.4 eq. silanolate groups, estimated) and without glass added in the reaction mixture in plastic tubes; monitored ions were indicated and representative mass spectra are shown in Fig. S1.

b Reaction progress *I*_P_/*I*_R_ represented by all the peak heights of the monitored product (P) *versus* the monitored residual reactant (R) as indicated in the schemes was analysed by nESI-MS after four hours of reaction; triplicate reactions were performed to estimate the average (two significant figures preserved) and the standard deviation.

c Acceleration (Accel.) factors were calculated (two significant figures) using the average reaction progress with glass *versus* without glass.

After 4 hours of incubation at room temperature, three replicates of solutions with glass microspheres and three replicates of controlled reaction mixtures were sampled using nESI-MS and from the signals of the monitored ions we calculated the ratios of the product formed *versus* the residual reactants. Clearly, the reactions progress much faster with glass microspheres (0.4 eq. silanolate groups) than do the controls. The acceleration factor (ratio of rates with and without glass) can be up to three orders of magnitude for certain types of reactions. The high sensitivity of the measurement enables us to study reactions of neutral reactants and still observe acceleration by glass microspheres as shown in entry 4.

Also, in this set of experiments, we found that no matter what solvent (H_2_O, MeOH, or ACN) was used, the phenomenon of enhanced kinetics by glass always exists. This motivated us to explore in more depth the chemistry induced by glass surfaces, aiming at understanding the mechanism and solvent effects on reaction acceleration by glass surfaces. Furthermore, the fact that some biomolecules such as acetylcholine and glutathione (entry 2 and 5) can be chemically degraded upon contact with glass when stored as a solution, stimulating our interest in the chemical degradation of a specific set of biomolecules–phospholipids – induced by glass surfaces.

### Mechanistic study of glass as a green catalyst by nESI-MS

**Fig. 2:**
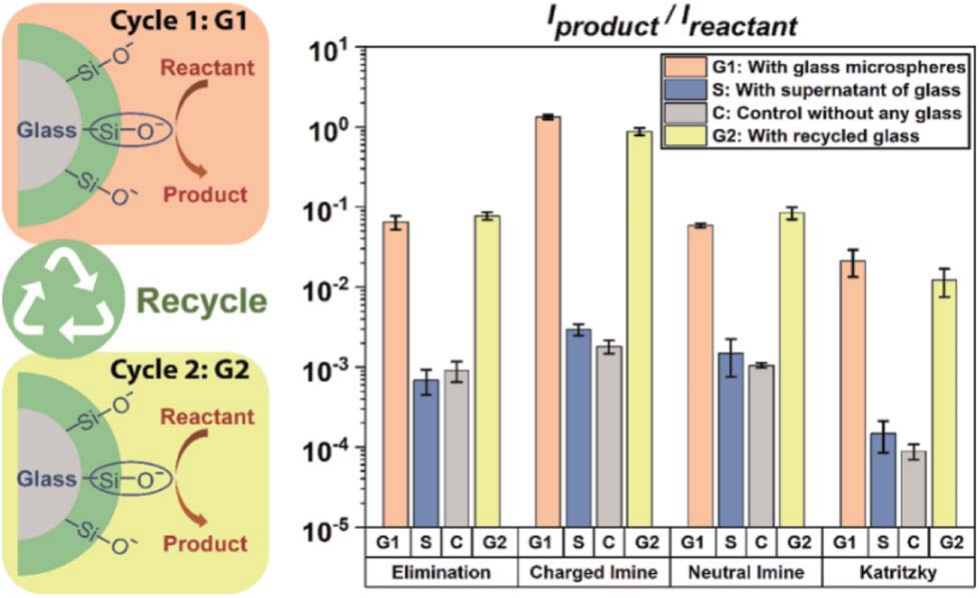
Scheme (left) and data (right) showing that glass microspheres can be easily recycled and can accelerate a second round of reactions, so acting as a ‘green’ heterogeneous catalyst. Reaction progress (at 50 μM) after 4 h with glass microspheres (G1 bar in orange: 0.4 eq. silanolate groups, estimated) and other conditions (S bar in blue: with supernatant of solution above glass microspheres added to the reaction mixture; C bar in grey: the control experiment without any glass microspheres or supernatant added; G2 bar in yellow: with recycled glass microspheres added) were compared using nESI-MS analysis (average of 3 replicates). Several order of magnitudes enhancement in reaction rates with glass microspheres added (both cycle 1 and cycle 2) and no increased in rate with supernatant added compared to the control were found for these four chemical reactions in acetonitrile: (1) elimination: elimination of HCl from 3-chloro-2-hydroxypropyl trimethylammonium; (2) charged imine: imine formation between Girard's reagent T and 2-pyridinecarbaldehyde; (3) neutral imine: imine formation between 3-(diethylamino)propylamine and 3-hydroxybenzaldehyde; (4) Katritzky: Katritzky reaction between 2,4,6-triphenylpyrylium and Girard's reagent T.

It is known from previous preliminary work^6^ that glass can be reused and acts as a ‘green’ heterogeneous catalyst for the Katritzky reaction in acetonitrile. Here we examined four reactions in acetonitrile, to test if such heterogeneous catalysis is a general phenomenon when using glass microspheres in an aprotic solvent. Excitingly, all four base-catalyzed reactions in acetonitrile showed similar trends to support the fact that glass microspheres can be easily reused as an excellent ‘green’ heterogeneous catalyst. As shown in Fig. 2, reaction rates increased by two orders of magnitude when glass microspheres were added under the experimental conditions for the E_2_ elimination reaction as well as for neutral imine formation, and by three orders of magnitude for the charged imine formation as well as in the Katritzky reaction of two positively charged reactants which showed virtually no product formation in a control experiment. After rinsing of the glass microspheres, they can be reused for all four reactions and their catalytic power (yellow bar in Fig. 2) is virtually unchanged. The supernatant test involves addition of the solvents in the glass microsphere solution into the reaction mixture, and the results showed that the kinetics were not altered compared to the control. This combination of phenomena showed that glass microspheres can be easily recycled without a significant reduction in their catalytic power in acetonitrile and that they can be used as general heterogeneous catalysts for base-catalyzed reactions. Representative mass spectra are shown in Fig. S2–S5.†

Besides these four base-catalyzed synthetic reactions, we also explored the solvolysis of acetylcholine in acetonitrile as shown in Fig. S6 and S7.† We found that the kinetics of this reaction in acetonitrile were little affected using glass microspheres (1.6-fold increase). This indicates that although silanolate groups at the glass surfaces have strong basicity they lack nucleophilicity and thus do not participate in the solvolysis reaction. This result suggests that the previously discussed glass promoted solvolysis in methanol (entry 2 in Table 1) is mainly due to the power of glass surface as a strong base to generate a better nucleophile (methoxide) by deprotonation of methanol; the effect is similar to that of adding sodium hydroxide to methanol but larger. To further test our hypothesis of deprotonation of solvent molecules by glass surfaces, we tested another amphiprotic solvent, water. Observation of the same product indicated the occurrence of nucleophilic attack by hydroxide on the carbonyl group (hydrolysis of acetylcholine). Moreover, the fact that there was acceleration when methanol or water (after contacting glass microspheres, *i.e.*, supernatant) was added, further supports the hypothesis that silanolate can act as a strong base to convert protic solvent molecules into anions which exist in the supernatant and so promote the reaction. This result broadens the scope of glass effects on chemical reactions since the silanolate groups not only affect base-catalyzed chemical reactions themselves as base catalysts but also act as a strong base to turn protic solvent molecules into powerful nucleophiles to affect nucleophilic reactions.

Because solvent molecules participate in E_2_ elimination reactions, we also investigated this reaction in detail for multiple solvents under different conditions as shown in Fig. S6 and S8.† Clearly, silanolate groups at glass surfaces in acetonitrile tend not to participate in solvolysis due to their poor nucleophilicity; however, acceleration of the E_2_ elimination reaction in acetonitrile still occurs by a factor of 71, due to their strong basicity. The results of elimination reactions in protic solvent showed similar supernatant effects to these seen in the case of acetylcholine described above. These findings lead to the conclusion that in such solvents both silanolate groups at glass surfaces and solvent anions produced by glass surfaces contribute to acceleration of base-catalyzed reactions. The relatively poor recovery of glass surfaces in protic solvents after recycling is not fully understood.

### Degradation of biomolecules induced by glass as studied by nESI-MS

The finding that biomolecules such as glutathione and acetylcholine can undergo significant amounts of chemical degradation when in contact with glass surfaces, raises awareness of the possible significance of the phenomenon considering that many important biomolecules are stored in solution in glass containers. Considering the importance of phospholipids in many practical aspects of chemistry^49,50^ including mRNA-based COVID-19 vaccine,^51^ we chose to study three types of phospholipids in detail: zwitterionic phosphocholine (PC) and zwitterionic phosphoethanolamine (PE) as well as negatively charged phosphatidylglycerol (PG).

**Fig. 3:**
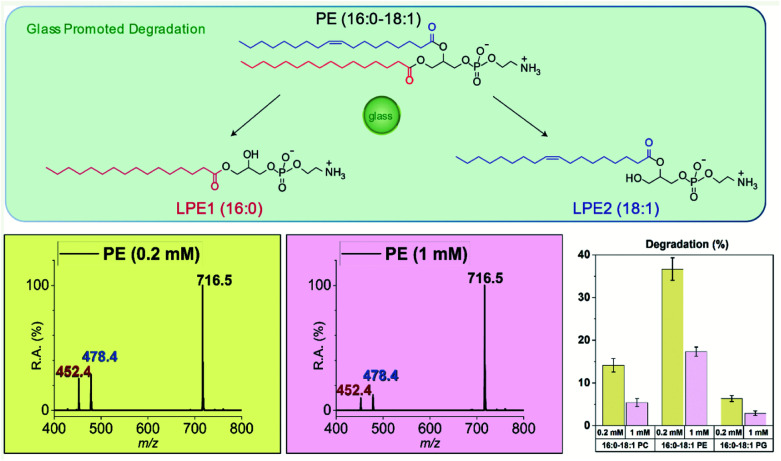
(Top) Scheme, (bottom left) representative nESI mass spectra (negative mode) with deprotonated PE signals labeled, and (bottom right) data showing that glass microspheres can promote degradation of lipids. Degradation of lipids after 48 h of incubation with glass microspheres at different concentrations: 0.2 mM of lipids (0.1 eq. silanolate groups, yellow) and 1 mM of lipids (0.02 eq. silanolate groups, purple). Average values for 3 replicate reactions were used. Larger percentages of degradation were found at lower lipid concentration for the three phospholipids stored in methanol: (1) 16:0–18:1 PC; (2) 16:0–18:1 PE; (3) 16:0–18:1 PG.

As shown in Fig. 3, 16:0–18:1 PE undergoes a significant amount of solvolysis at both ester chains in methanol upon incubation with glass microspheres, the same behavior as seen in the acetylcholine case. Because lipids of very low concentration are often used in the lipidomics studies, the concentration dependence on glass promoted degradation was investigated. After 48-h incubation of 1 mM 16:0–18:1 PE with glass microspheres (with 0.02 eq. silanolate groups, estimated), 17% of the PE was degraded into 16:0 LPE and 18:1 LPE and when the concentration of 16:0–18:1 PE was reduced to 0.2 mM (with 0.1 eq. silanolate groups, estimated) 37% of the PE was degraded. The same solvolysis reactions at both chains and similar concentration dependence were also observed in another zwitterionic phospholipid, 16:0–18:1 PC, and the negatively charged 16:0–18:1 PG. All three phospholipids studied were labile in contact with glass surfaces and when in contact with the same glass surface lower concentrations of phospholipids gave higher percentages of degradation. Representative mass spectra of PC and PG degradation are shown in Fig. S9 and S10† and the degradation percentages calculated from the signal intensities of both lysophospholipids *vs.* the sum of the signal intensities of lysophospholipids as well as the residual amounts of phospholipid are summarized in the bar chart in Fig. 3. These results should draw attention to possible deficiencies in current protocols involving storage of phospholipids in organic solvents in glass containers.

## Experimental

Reactants including (*S*)-(−)-(3-chloro-2-hydroxypropyl)trimethylammonium chloride (99%), acetylcholine chloride (≥99%), Girard's reagent T (99%), 2-pyridinecarbaldehyde (99%), 1,2,3,3-tetramethyl-3*H*-indolium iodide (98%), 3-hydroxybenzaldehyde (≥99%), 3-(diethylamino)propylamine (≥99%), l-glutathione reduced (≥98%), 2,4,6-triphenylpyrylium tetrafluoroborate (98%), *p*-anisidine (≥99%) and sodium hydroxide (pellets, semiconductor grade, 99.99% trace metals basis) were purchased from Millipore Sigma. Lipids such as 1-palmitoyl-2-oleoyl-*sn-glycero*-3-phosphoethanolamine (16:0–18:1 PE, >99%), 1-palmitoyl-2-oleoyl-*sn-glycero*-3-phospho-(1′-*rac*-glycerol) (sodium salt) (16:0–18:1 PG, >99%), and 1-palmitoyl-2-oleoyl-*glycero*-3-phosphocholine (16:0–18:1 PC, >99%) were purchased from Avanti Polar Lipids, Inc. Methanol and acetonitrile (Optima grade) were purchased from Fisher Scientific and ultra-pure water was from the Thermo Scientific™ Barnstead™ MicroPure™ water purification system (MicroPure UV ultrapure water system with UV-photo-oxidation). Reagents were dissolved in specific solvents to make stock solutions and diluted in the same solvent in polypropylene containers, then freshly mixed in stoichiometrically equal amounts to the desired initial concentrations just before the reaction. Soda lime glass microspheres (typical composition: SiO_2_: 60–72.5%; Na_2_O: 13.7–17%; CaO: 9.8–18%; MgO: 1–3%; Al_2_O_3_: 0.4–4%; FeO/Fe_2_O_3_: 0–0.2%; K_2_O: 0–0.1%; B_2_O_3_: 0.0%) were purchased from Thermo Scientific (NIST traceable mean diameter: certified mean diameter: 32.5 μm ± 1.2 μm; approximate number of glass microspheres: 2.3 × 10^7^ per gram; calibration batch: 9030-006; lot #230020) and after mixing with solvent, the solutions with glass microspheres were then mixed with pre-dissolved reactant solutions.

The reaction containers for the DESI experiments (1 mL Clear Glass Shell Vial, 8 × 30 mm, used without further treatment) were purchased from Analytical Sales and Services, Inc. The vials sat unstirred in Para-dox Aluminum Reaction Blocks (Parallel Synthesis/Optimization 96-Well Block Assembly) with a chemically compatible Teflon PFA sheet on the top, sealed by screwing the cover tightly with two silicone rubber mats for compression sealing. First, 15 mg glass microspheres were weighed and added into the reaction vessels, followed by addition of solvent and then addition of each reagent solution. After brief mixing of the solution at 1 mM, 15 μL of the solutions from the vials were transferred into a polypropylene 384-well plate (microplate, 384 well, V-bottom, natural; Greiner Bio-One North America, Inc.) with four replicate solutions from each vial. Thereafter 50 nL of solution in each well of the 384-well plate were pinned onto a DESI slide (microporous PTFE film – ZITEX G115 from Saint-Gobain Performance Plastics Corporation – manually glued on a Abrisa Technologies glass sheet) without delay with four replicate pinned spots per well and observed under a microscope to be dry right after pinning. DESI-MS experiments were performed, again without delay, with methanol as the spray solvent (2.75 μL min^−1^) and nitrogen as the nebulizing gas (150 psi). DESI-MS data was collected using a Thermo LTQ XL mass spectrometer with a Prosolia DESI 2D stage mounted on. The source parameters were as follows: capillary temperature 300 °C, capillary voltage +38 V, tube lens voltage +65 V, source voltage +4 kV. The automatic gain control was on with a maximum injection time of 100 ms. Mass spectra were recorded in the range from *m*/*z* 50–1000.

The Eppendorf safe-lock plastic tubes (2.0 mL, Eppendorf Quality™, colorless, polypropylene) were used as reaction containers in all the nESI-MS experiments and shut tightly with the cap to prevent solvent evaporation in bulk kinetic studies. The unstirred bulk kinetic study was made using 0.45 mL of reaction mixtures which were first thoroughly mixed at 50 μM (with *ca.* 15 mg glass microspheres if applicable) and then allowed to sit undisturbed at room temperature in the plastic tubes organized in vial racks followed by occasional sampling using 10 μL aliquots for nESI-MS. For glass recycle experiments, after analysis of the first round of reactions, reaction solution was carefully pipetting out and 1.5 mL of pure solvent was added to wash the used glass microspheres each time for three rounds until the ion signals relevant to reactions reached background levels in the pure solvents, and thereafter the second round of chemical reactions was performed.

Thick/standard wall borosilicate glass without filament (B150-86-10) was purchased from Sutter Instruments, cleaned by sonication in a mixed solvent (acetone : methanol : 2-propanol = 1 : 1 : 2; HPLC grade) and allowed to dry. The cleaned glass capillaries were then pulled into nESI capillaries with *ca.* 2 μm tip inner diameter using a Flaming/Brown micropipette puller (P-97 by Sutter Instruments). Non-accelerating conditions were achieved by using a short distance between the nESI sprayer tip and MS inlet (*ca.* 3 mm). During nESI-MS analysis, the electrode (stainless steel acupuncture needle, Beijing Zhongyan Taihe Medical Instrument Co., Ltd.) was in constant contact with the 10 μL aliquots in the capillary and between trials the needle was wiped using Kimwipes from Kimtech Science® with methanol to avoid carry-over between two rounds of sampling. No carry-over signal was detected between trials. The mass spectrometer used for mass analysis was a Thermo LTQ instrument. The source parameters were as follows: capillary temperature 200 °C, capillary voltage +8 V (−22 V for negative mode), tube lens voltage +40 V (−45 V for negative mode), source voltage +1.5 kV (−1.5 kV for negative mode, applied on the electrode by a clip). The automatic gain control was on with a maximum injection time of 100 ms (250 ms for negative mode). Mass spectra were recorded in the mass range from *m*/*z* 50–1000. An average of 100 scans was used in each trial. Peak height ratios were used for kinetic calculations. All experiments were performed in triplicate and standard deviations were calculated to determine uncertainties.

## Conclusions

With the aid of high-throughput DESI-MS, systematic screening of accelerated chemical reactions at solid/solution interfaces has been carried out, providing evidence that glass surfaces accelerate various base-catalyzed chemical reactions. The reaction types affected include (E_2_) elimination, solvolysis (hydrolysis and transesterification), condensation (imine formation, Katritzky reaction as well as Knoevenagel condensation), and oxidation (of thiol to disulfide). This systematic study has greatly broadened the scope of substrates affected by glass reactions from positively charged molecules to neutral molecules, zwitterionic molecules as well as negatively charged molecules. In a detailed mechanistic study, we demonstrated using nESI-MS that in aprotic solvents such as acetonitrile, glass can be used as a general heterogeneous catalyst; it is also ‘green’ and can be easily recycled simply by rinsing.

We also provided evidence that glass surfaces can act as strong bases and convert protic solvents to their conjugate bases which then act as base/nucleophile to promote chemical reactions. Last but not least, glass surfaces are also found to degrade chemicals, notably phospholipids, and the storage of base/nucleophile-labile biomolecules in glass containers should therefore be avoided. Increasing extents of degradation at low concentration, as shown in the case of phospholipid degradation, should be of broad interest considering the significance of lipid studies in bioanalytical science.

## Author contributions

Y. Li led the conceptualization and writing of the original draft. Y. Li and K.-H. Huang contributed equally to methodology, data curation, investigation, validation, visualization, and formal analysis. N. M. Morato contributed to the software for the formal analysis of the DESI-MS data and supported data curation. R. G. Cooks provided key advice and context and supervised the study. All authors contributed to the writing of the manuscript.

## Conflicts of interest

There are no conflicts to declare.

## Supplementary Material

SC-012-D1SC02708E-s001
