# Supplementary material for: Glass surface as strong base, ‘green’ heterogeneous catalyst and degradation reagent
Source: Chem Sci. 2021 Jun 23;12(28):9816–22. doi: 10.1039/d1sc02708e (PMC8294000; doi:10.1039/d1sc02708e)
Supplement: SC-012-D1SC02708E-s001 [file SC-012-D1SC02708E-s001.pdf]

Electronic Supplementary Material (ESI) for Chemical Science.
This journal is © The Royal Society of Chemistry 2021

Supporting Information

Glass surface as strong base, 'green' heterogeneous catalyst and degradation reagent

Yangjie Li,† Kai-Hung Huang,† Nicolás M. Morato and R. Graham Cooks*

Department of Chemistry, Purdue University, West Lafayette, Indiana 47907, United States

e-mail:cooks@purdue.edu

Table of Contents

Table of Contents 1
Representative mass spectra of glass-promoted chemical reactions 2
Representative mass spectra and plots of systematic glass acceleration mechanistic studies 3
Representative mass spectra of glass-promoted degradation of biomolecules 10

Representative mass spectra of glass-promoted chemical reactions

Figure S1 shows representative mass spectra of glass-promoted chemical reactions with results summarized in Table 1 as well as in the table-of-content figure.

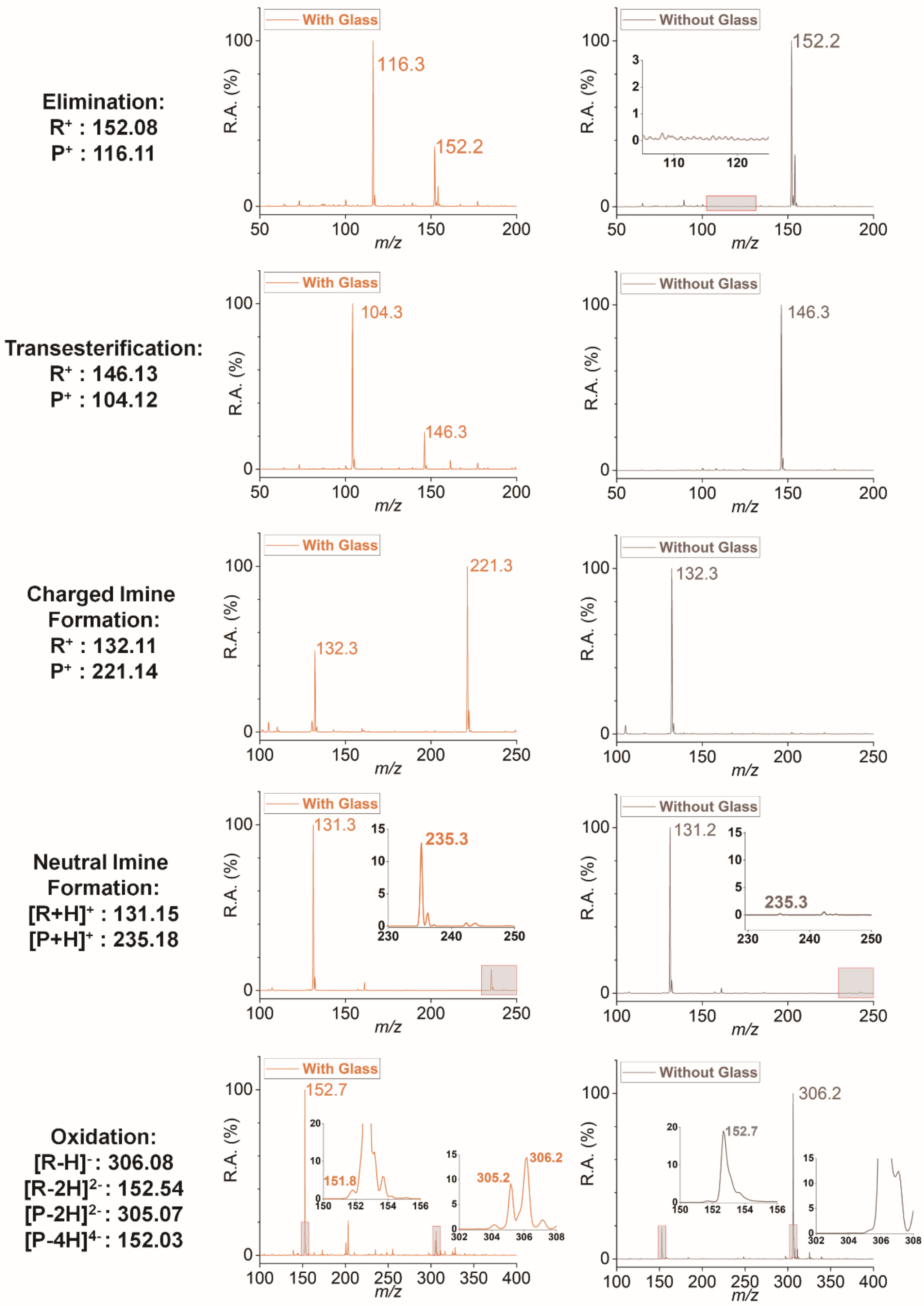

Figure S1. Representative mass spectra of glass-promoted chemical reactions listed in Table 1 with glass microspheres (orange) and without glass microspheres (grey). Reactions are examined by nESI after four hours under ambient conditions.

Representative mass spectra and plots of systematic glass acceleration mechanistic studies

Figure S2-S5 show representative mass spectra of four base-catalyzed chemical reactions in acetonitrile under different conditions; data from three replicates were summarized in Figure 2. From the results, it is clear that in an aprotic solvent such as acetonitrile glass can be used as a general heterogeneous catalyst and a ‘green’ one which can be easily recycled using simple rinsing.

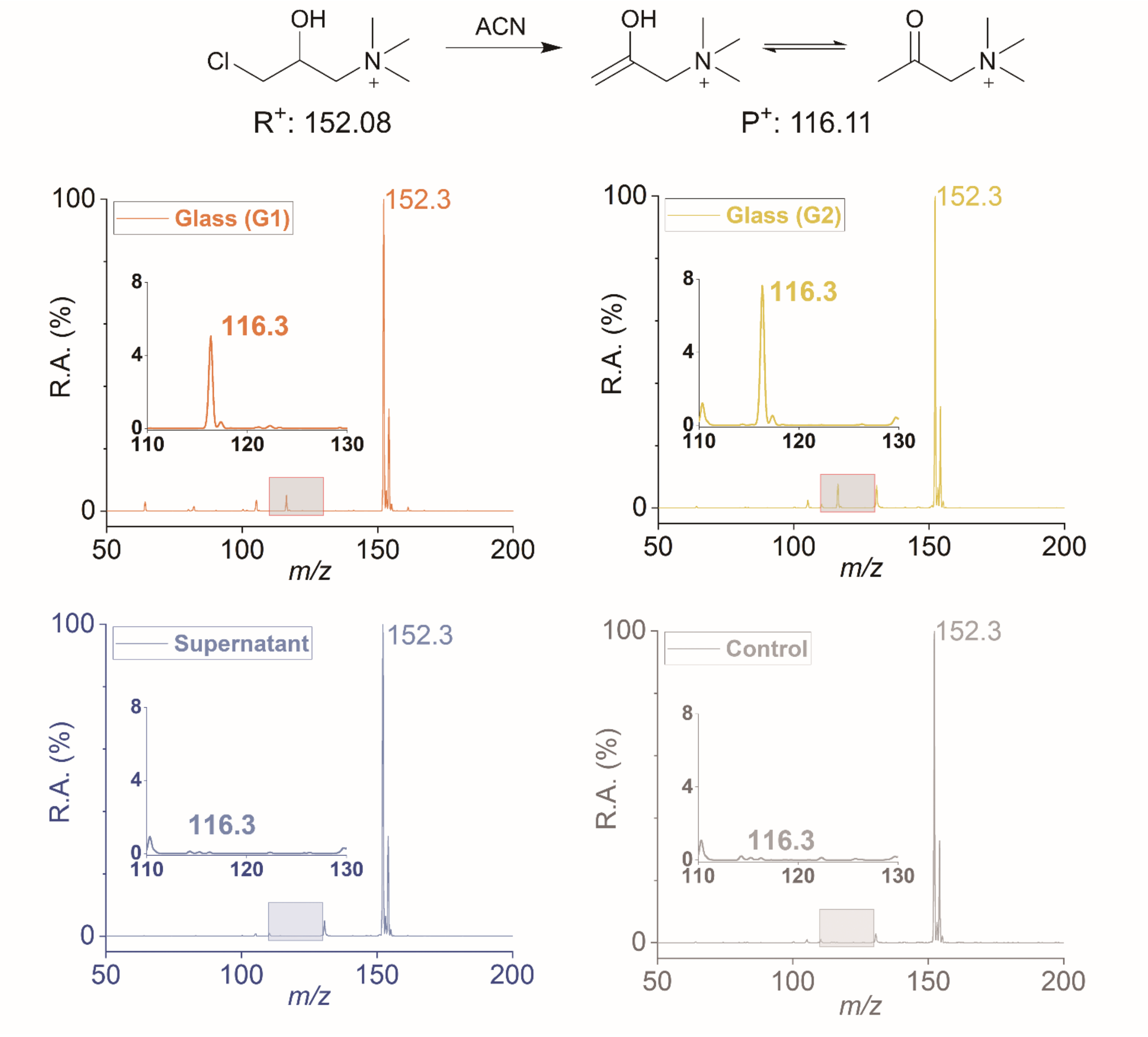

Figure S2. Reaction scheme and representative nESI mass spectra showing the progress of elimination reaction in acetonitrile (at 50 μM) after 4 h under following conditions: with glass microspheres with 0.4 eq. silanolate groups estimated at the surfaces (in orange), with recycled glass microspheres added (in yellow), with supernatant of solution above glass microspheres added in the reaction mixture (in blue), and the control experiment without any glass microspheres or supernatant added (in grey).

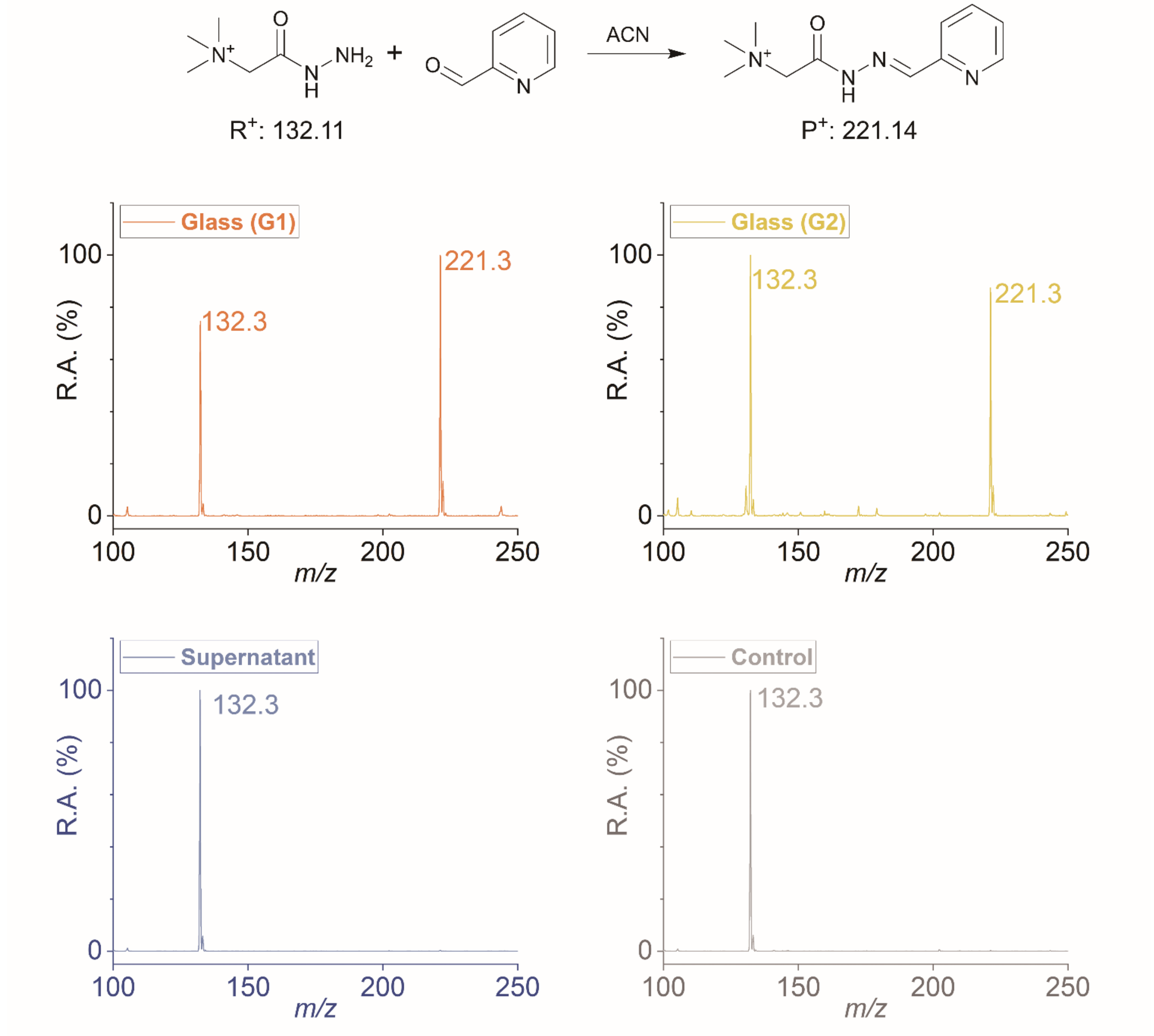

Figure S3. Reaction scheme and representative nESI mass spectra showing the progress of charged imine formation in acetonitrile (at 50 μM) after 4 h under the following conditions: with glass microspheres with 0.4 eq. silanolate groups estimated at the surfaces (in orange), with recycled glass microspheres added (in yellow), with supernatant of solution above glass microspheres added in the reaction mixture (in blue), and the control experiment without any glass microspheres or supernatant added (in grey).

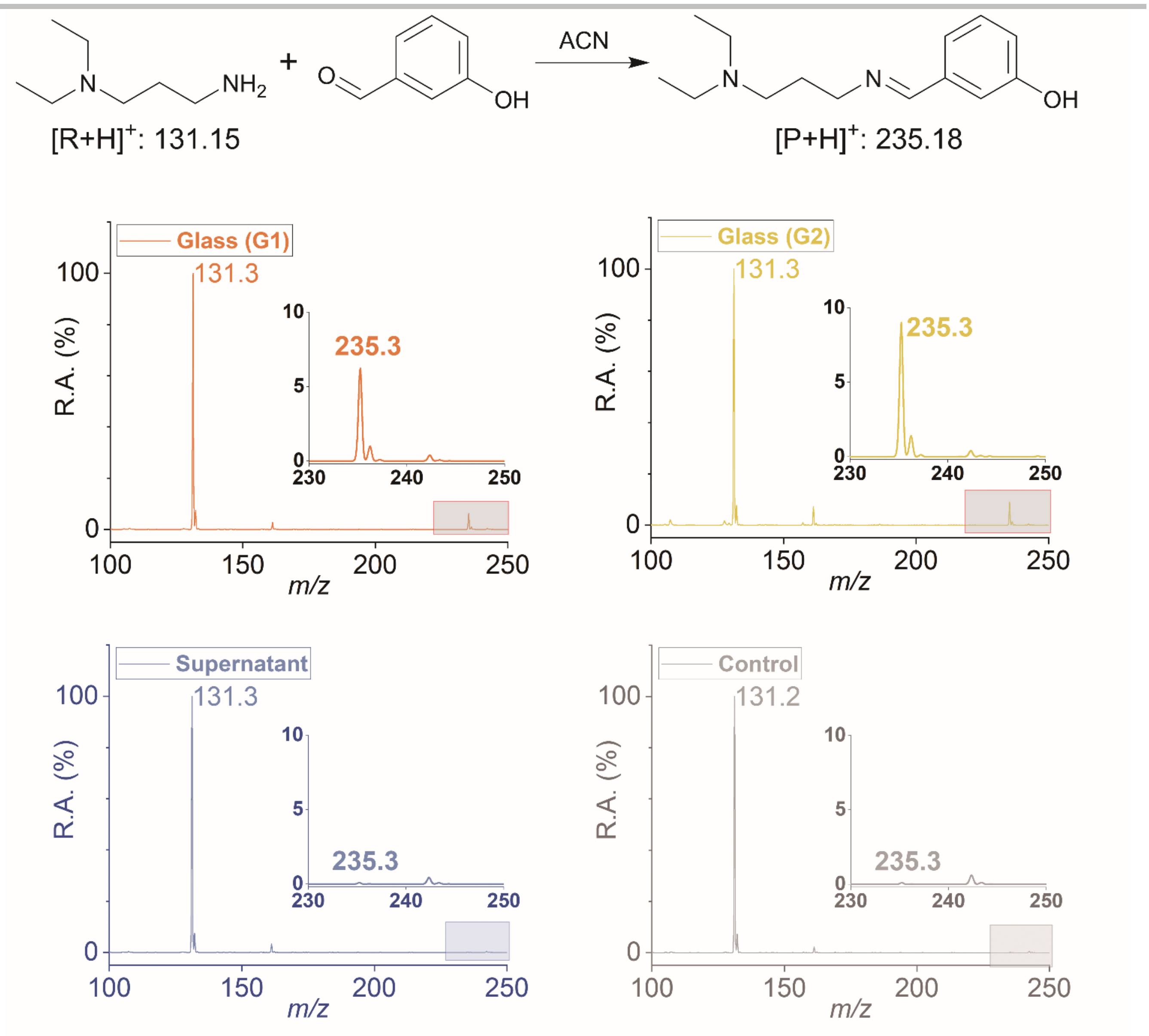

Figure S4. Reaction scheme and representative nESI mass spectra showing the progress of neutral imine formation in acetonitrile (at 50 μM) after 4 h under the following conditions: with glass microspheres with 0.4 eq. silanolate groups estimated at the surfaces (in orange), with recycled glass microspheres added (in yellow), with supernatant of solution above glass microspheres added in the reaction mixture (in blue), and the control experiment without any glass microspheres or supernatant added (in grey).

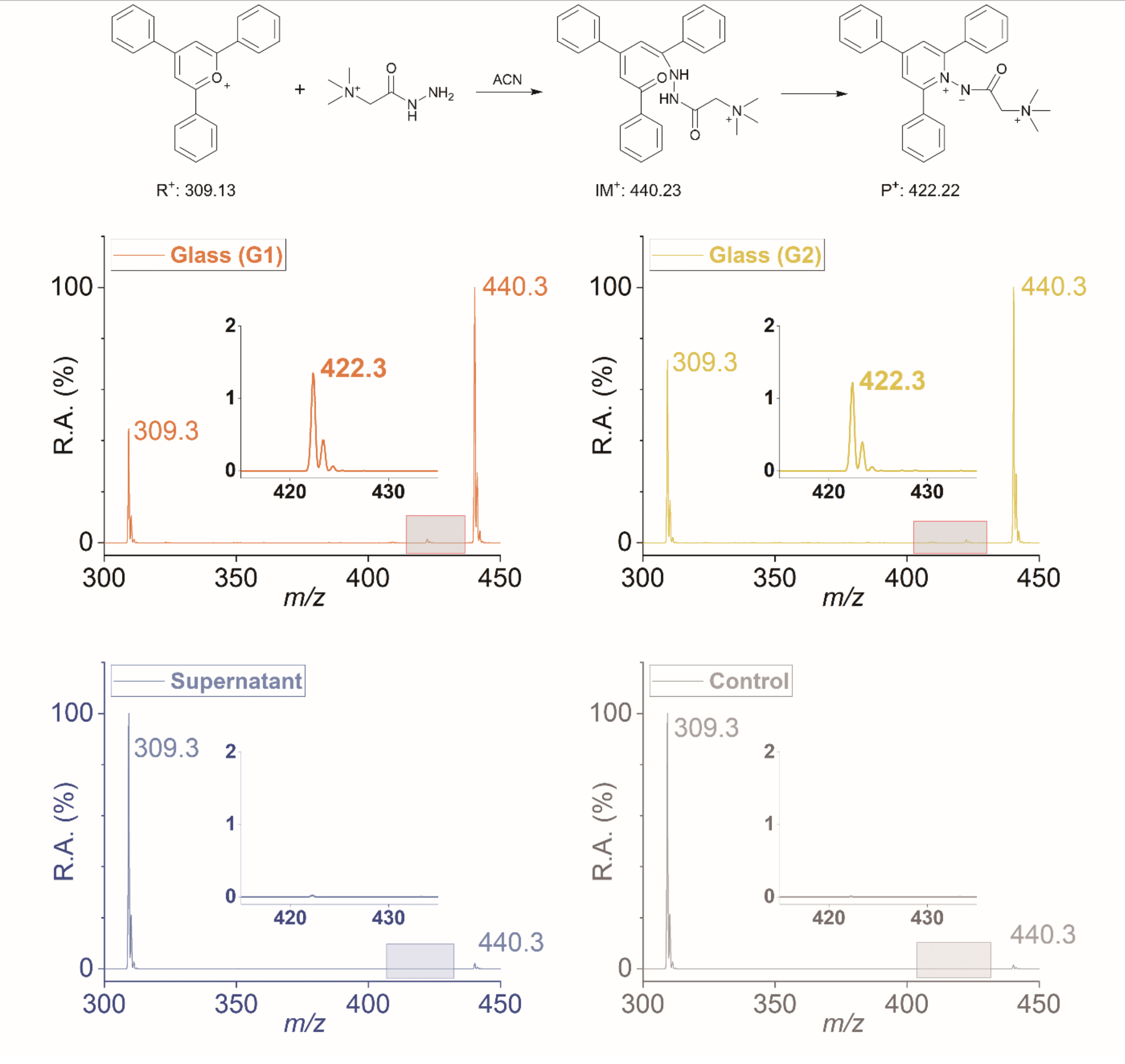

Figure S5. Reaction scheme (with the charged intermediate represented by IM^+) and representative nESI mass spectra showing the progress of Katritzky reaction of two positively charged reactants in acetonitrile (at 50 μM) after 4 h under the following conditions: with glass microspheres with 0.4 eq. silanolate groups estimated at the surfaces (in orange), with recycled glass microspheres added (in yellow), with supernatant of solution above glass microspheres added in the reaction mixture (in blue), and the control experiment without any glass microspheres or supernatant added (in grey).

As shown in Figure S6, we studied two different reactions in three different solvents. Representative mass spectra from these experiments are shown in Figure S7 and S8.

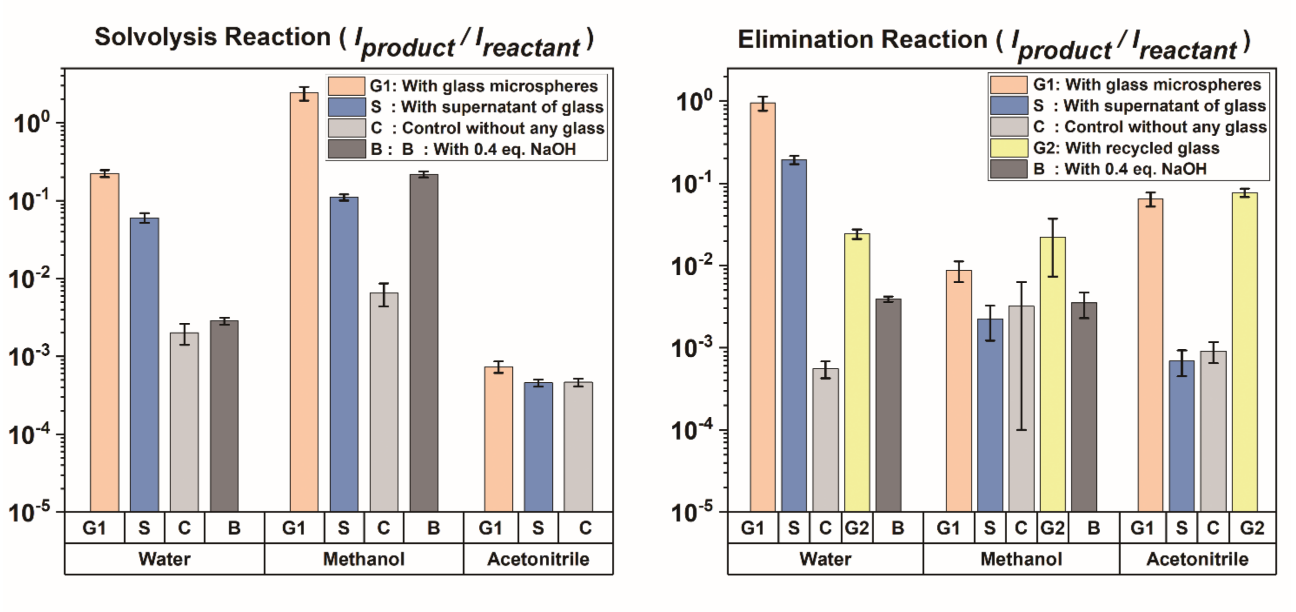

Figure S6. Column plots for the reaction progress (at 50 µM) for solvolysis of acetylcholine and elimination of hydrogen chloride from 3-chloro-2-hydroxypropyl trimethylammonium after 4 h in different solvents. Conditions which were investigated included: 0.4 eq. silanolate groups (estimated) as added glass microspheres; supernatant of the solution above glass microspheres added to the reaction mixture; control experiment without any glass microspheres or supernatant added; with addition of recycled glass microspheres; 0.4 eq. sodium hydroxide added. Data were acquired using nESI-MS for analysis (average value of 3 replicate analysis used), and values were calculated as the ratio between signal intensity of product to signal intensity of reactant.

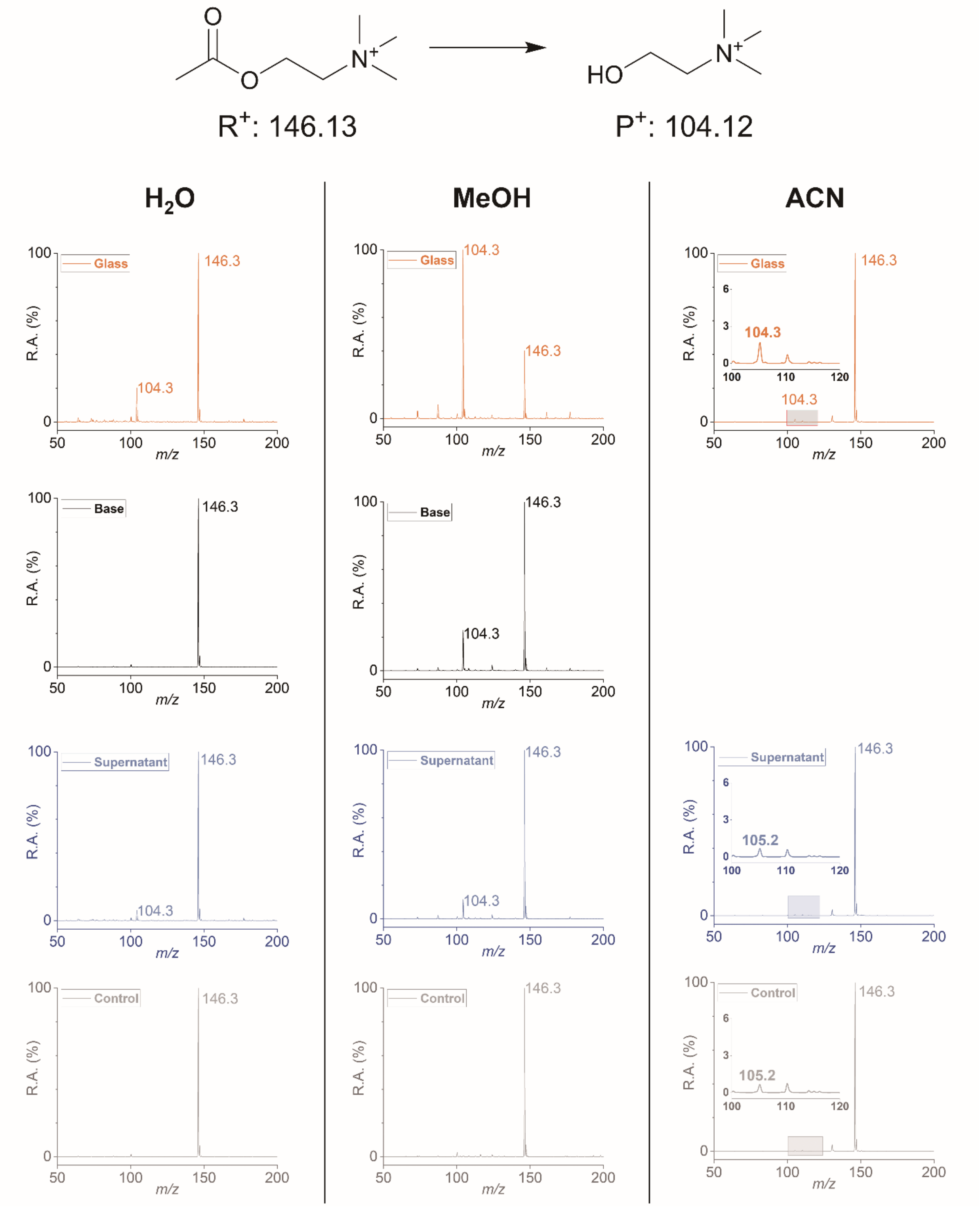

Figure S7. Representative nESI mass spectra showing the progress of the solvolysis of acetylcholine in three different solvents (at 50 μM) after 4 h under following conditions: with 0.4 eq. surface silanolate groups (est.) added glass microspheres (in orange), with 0.4 eq. sodium hydroxide added as a standard base comparison (in black), with supernatant of solution above glass microspheres added in the reaction mixture (in blue), and control experiment without any glass microspheres or supernatant added (in grey).

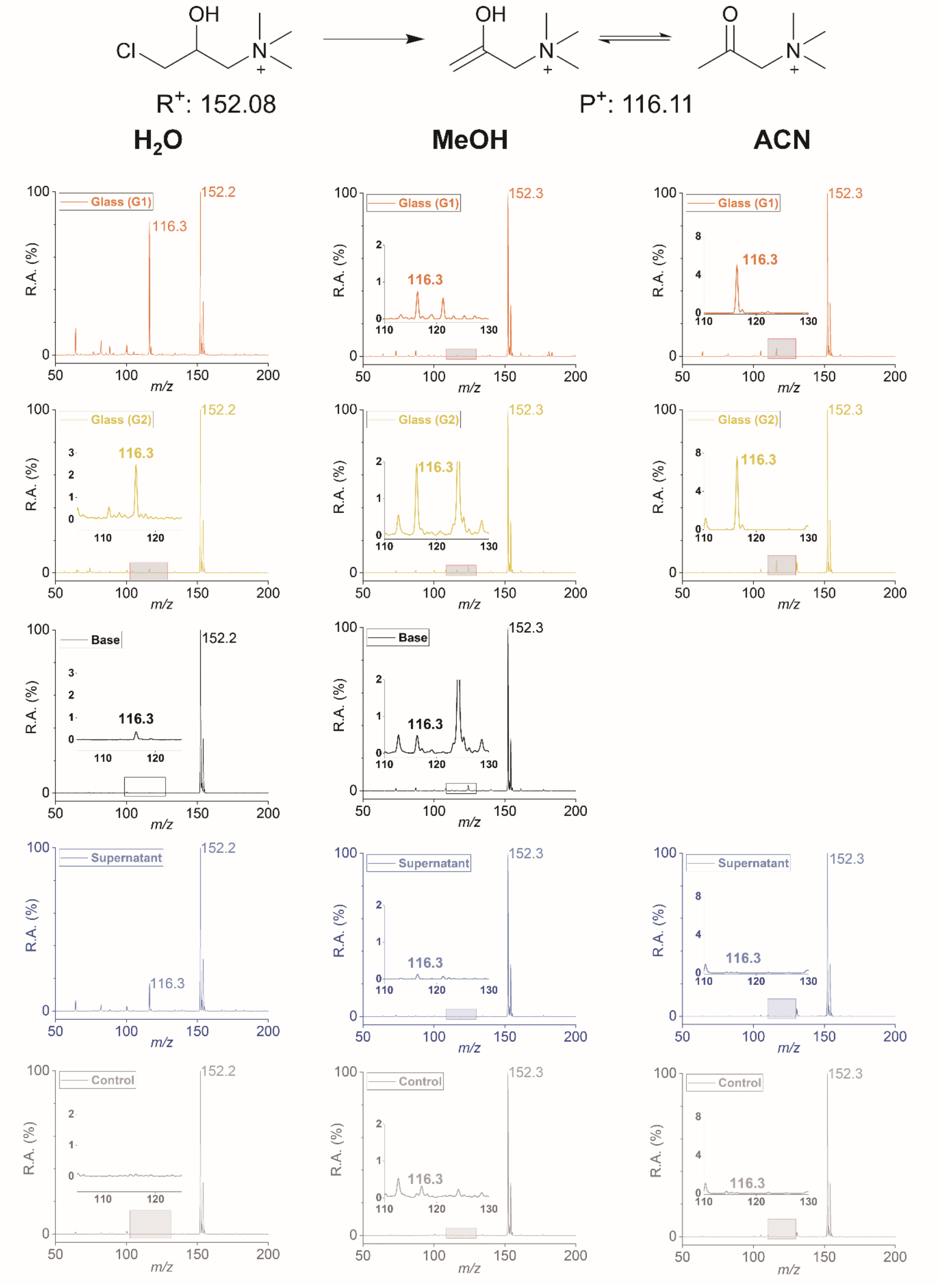

Figure S8. Representative nESI mass spectra showing the progress of elimination reaction in different solvents (at 50 μM) after 4 h under the following conditions: with 0.4 eq. surface silanolate groups (est.) added glass microspheres (in orange), with recycled glass microspheres added (in yellow), with 0.4 eq. sodium hydroxide added as a standard base comparison (in black), with supernatant of solution above glass microspheres added in the reaction mixture (in blue), and control experiment without any glass microspheres or supernatant added (in grey).

Representative mass spectra of glass-promoted degradation of biomolecules

Figures S9 and S10 show representative mass spectra of glass-promoted degradation of the two phospholipids with results summarized in the bar chart of Figure 3.

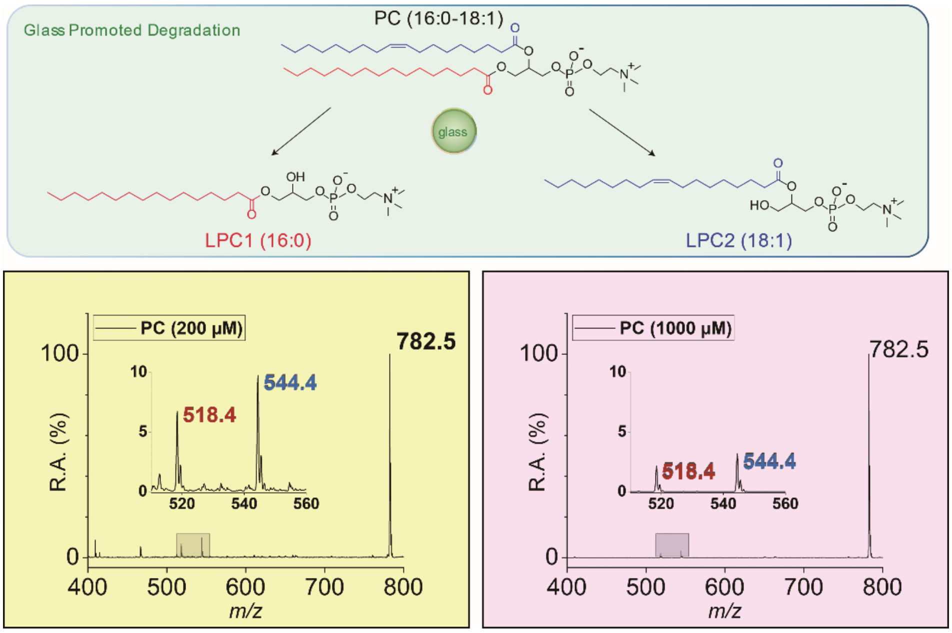

Figure S9. (top) Scheme and (bottom) representative nESI mass spectra (positive mode) of glass promoted degradation of PC after 48 h of incubation with glass microspheres at different concentrations: 200 μM of PC (0.1 eq. silanolate groups estimated, yellow) and 1000 μM of PC (0.02 eq. silanolate groups estimated, purple).

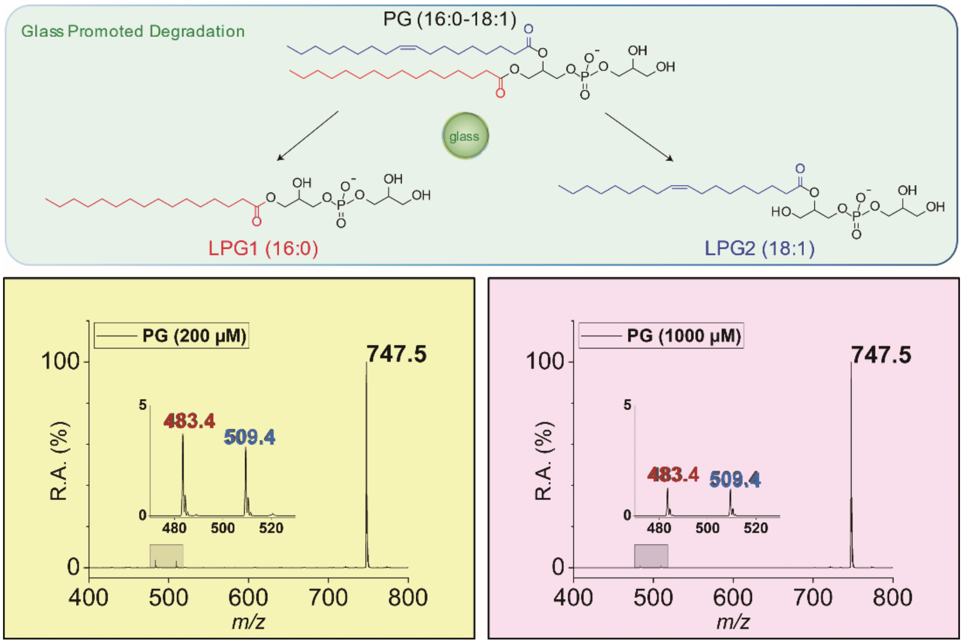

Figure S10. (top) Scheme and (bottom) representative nESI mass spectra (negative mode) of glass promoted degradation of PG after 48 h of incubation with glass microspheres at different concentrations: 200 μM of PG (0.1 eq. silanolate groups estimated, yellow) and 1000 μM of PG (0.02 eq. silanolate groups estimated, purple).
